# How to build large, multi-scale, functional brain models

**DOI:** 10.1186/1471-2202-15-S1-A1

**Published:** 2014-07-21

**Authors:** Chris Eliasmith

**Affiliations:** 1 Department of Systems Design Engineering, University of Waterloo, Waterloo, Ontario, N2L 3G1 Canada

## 

Recently, several large-scale brain models have been presented, including those from the Human Brain Project and IBM's Synapse Project. However, these large, complex models do not exhibit interesting psychological (i.e., motor, perceptual, and cognitive) behaviors. Consequently, they are difficult to compare to much of what we know about the brain. In this talk, I describe the methods (e.g., the Neural Engineering Framework) and tools (e.g., Nengo (http://nengo.ca)) used to construct what is currently the largest *functional* brain simulation. This model is called the Semantic Pointer Architecture Unified Network (Spaun) and uses 2.4 million spiking neurons organized to respect known anatomical and physiological constraints. I demonstrate the variety of behaviors the model exhibits and show that it is similar in many respects to human and animal behaviour. I show how Spaun allows comparison of the model to data across scales and across measurement modalities (e.g. spike trains, reaction times, error rates). I argue that constructing such large-scale simulations that permit this broad range of comparison to data is critical for advancing our understanding of neural and cognitive function, and I suggest that it helps to unify our understanding of how the mind works.

